# Detect pre-cancerous tongue lesions for early oral cancer diagnosis using deep learning algorithm

**DOI:** 10.1038/s41598-025-25925-1

**Published:** 2025-11-25

**Authors:** T. Benil, Raji Krishna, Tulasi Prasad Sariki, P. Yashika, Saanjhi Saraogi, Sakshi Saraogi

**Affiliations:** 1 School of Computer Science and Engineering, Vellore Institute of Technology (VIT), Chennai, Tamil Nadu 600127 India; 2 School of Electrical Engineering, Vellore Institute of Technology (VIT), Chennai, Tamil Nadu 600127 India

**Keywords:** Oral cancer, Tongue lesions, Automatic image detection, Deep learning, Convolutional neural networks, Cancer, Diseases

## Abstract

Precancerous tongue lesion is a prevalent, complex, and highly perilous kind of cancer. The tumour might be in the salivary glands, tonsils, neck, cheek, and mouth. Oral Cancer (OC) is commonly identified in advanced stages due to the limited accuracy of available screening methods for early detection despite their significant potential to reduce mortality rates. The study exclusively examines lesions that specifically manifest on the tongue. This work demonstrates that one of the deep learning (DL) such as convolutional neural networks (CNN) based models employed are novel in their capacity to effectively identify OC, primarily due to the limited research conducted in this field. The research utilizes a specifically created dataset due to the absence, to the best of our knowledge, of any existing information on tongue lesions occurring in the oral cavity. The research recommends using various methods, such as DenseNet121, DenseNet169, DenseNet201, MobileNet, MobileNetV2, VGG16, VGG19, ResNet50, EfficientNetV2B0, EfficientNetV2B1, EfficientNetV2B2, EfficientNetV2B3, Inception, AlexNet, and transfer learning, to enhance our ability to diagnose OC. The effectiveness of the enhanced technique is assessed based on customized data. The study’s input parameters consist of a portrait of the patient’s tongue. according to the assessed outcomes of training precision, validation precision, training loss, and validation loss. The outcome indicated that VGG16 achieved the highest performance based on the given parameters. It demonstrated a vital training accuracy of 97.66% and a commendable validation accuracy of 89.06%. The study’s Clinical trial number not applicable.

## Introduction

**Fig. 1:**
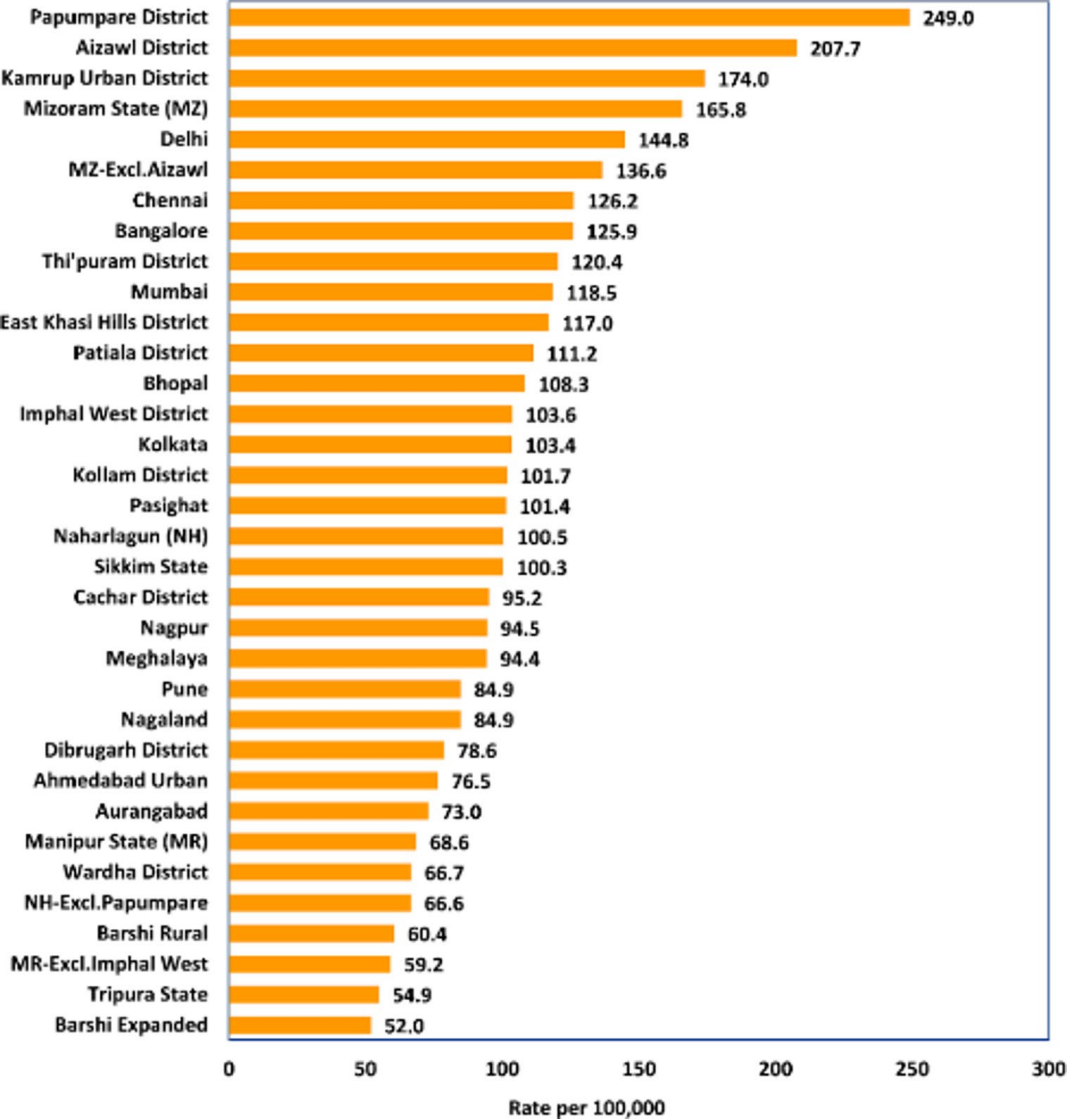
Distribution of oral cancer across India.

Mouth cancer is also known as oral cancer (OC).It is a type of malignant tumor that grows in the tissues of the mouth or throat. It falls under the largest group of head and neck cancers primarily affecting squamous cells in the tongue, lips, and mouth. Figure 1 shows that India is responsible for nearly one-third of all oral cancer cases globally. Terrifyingly about 30% of all cancers in India are oral cancers, underlining the danger of this public ailment.

Regular dental exams are necessary to prevent and detect OC, a common disease. This study proposes a novel approach to early detection and diagnosis using deep neural networks to monitor complex patterns automatically. OC becomes intractable as cells proliferate and invade neighboring tissues. Researchers have found that finding a small number of non-reproductive cells in oral tissue can help find ulcers in their early stages. Deceased cells may be found in the body or isolated regions at the site of metabolic activity. No special tools are needed to see the mouth. Specialists use their visual knowledge of malignant tumors to examine and diagnose mouth cancer in clinical practice. OC lesions usually appear as white patches with crimson patches or, in rare cases, as white-red stains. Often, the mucosal surface becomes progressively irregular, rough, and ulcerated. However, untrained medical staff may misinterpret these visual patterns as ulceration or other oral mucosal membrane disorders. No vision-based OC detection method has been scientifically validated. Oral biopsies detect OC. However, they are time-consuming and rarely available in primary care or communities, especially in poor countries.

According to reports of National Institute of Health (NIH) reports OC is a serious health issue that causes 177,384 deaths per year and is most common in developing and middle-income nations^1^. Transfer learning-based hexad Deep Convolutional Neural Network (DCNN) models were tested on a small dataset of medically classified photos. Initial results employing an Artificial intelligence (AI) enhanced physician ensemble model reveal that two DCNNs can automatically screen tongue lesions with classification performance close to humans^2^. Present and evaluate a new automated approach for identifying oral squamous cell cancer (OSCC) using CLE images and DL technologies^3^. The methodology is distinguished from surface texture feature-based machine intelligence techniques, which represent modernization at the time. It examines how well a CLE picture progression method can identify images of oral cavity OSCC lesions from 4 locations. The results show that this strategy outperforms modernized methods in CLE image recognition. A cascaded CNN approach to automatically identify oral cavity squamous cell carcinoma (OCSCC) from photographic pictures^4^. The study determined that using a DL algorithm enables automated detection of OCSCC, offering a fast, painless, cost-effective, and practical approach. The results obtained are comparable to those of human experts, suggesting that this method can be employed as a clinical tool for rapid screening, early detection, and assessment of cancer treatment efficacy. The metrics evaluated include the area under the receiver operating characteristic curves (AUCs), accuracy, and sensitivity. These metrics are assessed on the internal, external, and clinical validation datasets.

Three approaches are utilized to produce characteristics: wavelet features, Zernike Moment, and bag histogram of directed gradients^5^. The attributes were then categorized using the CNN classifier. The suggested technique was evaluated using error, recall, and accuracy rates. According to the evaluation, ABC, PSO, and CNN work better together to identify OC. To create an automated technique to identify mouth cancer early^6^. First and foremost, many carefully characterized oral lesions must be gathered. This paper presents an innovative way of categorizing OC photographs using automated algorithms. The project develops a computerized identification method for deep neural networks and machine learning. Dynamic Bayesian Networks (DBN), Artificial Neural Networks (ANN), Decision Trees (DT), Support Vector Machines (SVM), and K-Nearest Neighbour Algorithm classification models are built using the original data. These models automate OC detection and categorization^7^.

Pathologists evaluated tiny biopsy pictures for OC^8^. This procedure involves boosting microscopic pictures and converting RGB to LAB color space. K-means clustering groups colors. The threshold value classifies microscopic biopsy images as malignant or benign. Images processed using CNNs identify OC^6^. This investigation shows that background and bone components may be efficiently separated, helping doctors automate OC detection and diagnosis. An effective and direct-centered oral cavity imaging approach was presented^9^. This approach was utilized to collect medium-sized oral data on five illnesses and a testing procedure that decreased portable phone camera picture alterations. A modern DL network improves the suggested OC detection approach’s image classification, treatment planning, synthesis, segmentation, and DL algorithms^10^. The study also explores how this method might improve precision medicine and future OSCC treatments using DL technology.

Domain that uses machine learning on victim datasets^11^. Next, apriori categorization is used. Our software program for healthcare uses data mining and extraction for predictive approaches, classification rules for OC prediction, and association rules to detect linkages among OC features. Data mining uses association rule mining to find latent correlations between attributes. It finds stringent database rules. Validated oral endoscopic images for deep neural network tongue cancer detection^12^. The study retrospectively collected 12,400 verified endoscopic images. Different CNN architectures were used to create DL models to predict malignancy. The verified endoscopic image collection helped the DL model detect tongue cancer.

To assess multiple imaging modalities designed for OC screening and various image processing and machine learning techniques available for OC detection^13^. Various texture analysis techniques have been investigated, including Gray-Level Co-Occurrence Matrix (GLCM), Glucose Lymphocyte Ratio (GLR), Gabor features, fractal features, and local binary patterns. Research has also examined how various color attributes can be utilized to assess oral lesions. An efficient DL and feature selection technique employing the Alex net model has been used to get the best results in diagnosing OC^14^. An initiative to develop an automated system employing DL to diagnose oral lesions^15^. An ensemble DL model has been developed that integrates the strengths of Resnet-50 and VGG-16. The proposed ensemble DL model assists in distinguishing oral lesions from digital color images as benign or malignant. The ensemble model’s performance surpasses that of the individual models. To evaluate the efficacy of optical instruments for oral examination. This study included 314 patients who received optical tests at Tokyo Dental College from 2014 to 2018^16^. Subjective and objective assessments were employed to interpret images obtained via fluorescence viewing. Data mining technologies using AI algorithms, such as ANN and Multi-Layer Perceptron (MLP), have shown efficacy in scientific research for the diagnosis, staging, and treatment planning of OC. Furthermore, genomic data and DNA microarray methods, along with genetic algorithms and tools like DBN and SVM are extensively used in the early identification and monitoring of OC growth^17,18^. Conventional machine-learning techniques are categorized into supervised and unsupervised methods. The supervised approach depends on the trained machine learning model to verify the inputs and outputs that serve as the model’s ground truth against which the diagnostic input is evaluated^19^. On the other hand, unsupervised approaches use machine learning models that do not depend on predetermined values. Instead, they use data or specimens to find common latent characteristics through extraction and mining. Nonetheless, the thorough integration of AI in OC diagnostics has encountered several challenges. Issues about data privacy and security emerge while handling sensitive patient information. Moreover, the vast array of datasets pertinent to AI model training might provide a significant obstacle^20,21^. Moreover, a danger exists associated with excessive dependence on AI, which may replace the physician in diagnostic processes. Enhancing the accessibility of cost-effective AI technology for healthcare professionals globally is essential, especially in low-resource regions where the incidence of OC may be most pronounced^22^. The primary issue is that incorporating AI functionalities into current healthcare systems sometimes poses technological and logistical difficulties. Medical workers must undergo extensive training and education to use it effectively^23,24^. Managing the previously mentioned research gaps is essential for medical innovation. Our study builds on this establishment by proposing a deep neural network model designed for detecting precancerous tongue lesions. Unlike traditional handcrafted methods, our approach uses Convolution Neural Networks to automatically learn distinguishing features from images. It highlights the importance of explainability tools like saliency maps and Grad-CAM. These tools can enhance clarity and clinical trust, which are important for adoption in real world healthcare setting.

Artificial intelligence holds great potential in overcoming current limitations. Specifically, AI-based technologies provide a new paradigm for oral cancer screening by utilizing advanced image analysis and Deep Learning models to improve diagnostic accuracy and efficiency, thereby supporting and advancing the physician’s role. However, the poor explainability of DL models, sometimes referred to as ‘black boxes’. Therefore, DL-based technology for oral cancer screening is promising but presents challenges to overcome. Other considerations include issues related to data collection and the explainability of DL models. Regarding data collection, the consideration of what devices to use is paramount. Traditional devices, like smartphones, are inexpensive and simply accessible, helping to scale up devices for large-scale screening programs^25^. A hybrid method for the explainable classification of oral lesions, which combines a Deep Learning model with Case-Based Reasoning (CBR). This is a method of problem solving often used by physicians and involves the classification of a new case according to similarities with previously seen instances. In the proposed diagnostic system, each case is an oral lesion, and the corresponding class is the disease or condition explaining the lesion^26^.

Although several investigators have used deep learning for oral cancer detection, most of those studies emphasized oral cavity lesions or cancers in general without focusing on the tongue, which has specific clinical properties and risks. The present study is unique in that it focuses on pre-cancerous lesions located solely on the tongue and uses a particular dataset we compiled because data on these lesions are not available publicly. We believe that using multiple of the latest convolutional neural network architectures with custom evaluation based on the collected dataset highlights something novel and practically relevant in the early detection of tongue lesions, and the main goal is to develop a deep learning-based framework for the early detection of precancerous tongue lesions. By enhancing early diagnosis, this work provides clinicians with a cost-effective, non-invasive, and scalable solution that can significantly improve patient results.

This paper is organized as follows. In Sect. 2, the basic structure of proposed system is discussed, the implementation of the CNN algorithm for Oral Cancer in mouth is presented. In Sect. 3, discusses the simulation results using CNNs algorithms. Finally, the conclusion and future scope are discussed in Sect. 4.

## Proposed system

**Fig. 2:**
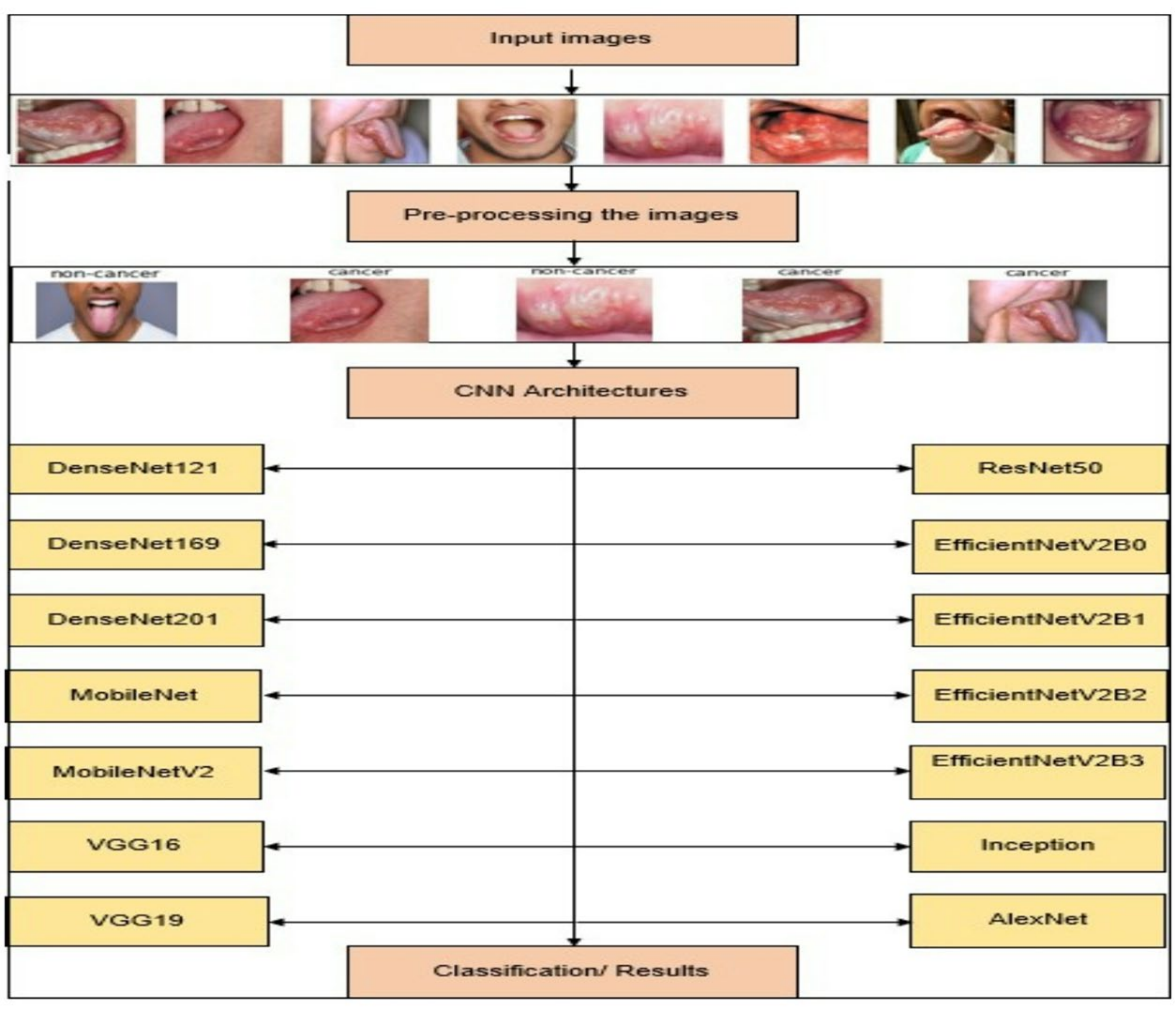
Block diagram for the proposed system.

The prompt identification of OC in its initial stage significantly improves survival rates. There has been a growing interest in using CNN model in diagnostic medicine. This research sought to objectively evaluate the existing data about CNN efficacy in diagnosing OC. Particular attention was devoted to CNN diagnostic precision and capacity to detect the early stages of OC. Figure 2 illustrates the block diagram of the proposed system.

### Implementation of CNN model for oral cancer in mouth

#### The data pre-processing

The step-by-step process of visualizing how oral tongue lesion data is collected, prepared, and preprocessed with the following significant steps given: (i) Data Collection Overview, (ii) Data Acquisition Protocol, (iii) Metadata Collection, (iv) Data Cleaning.

#### Data collection

The goal is to gather high-quality, annotated images and other modalities representing oral tongue lesions under various clinical conditions with the help of multiple sources.

#### Data acquisition protocol

 To achieve consistent and reproducible images, with standardized imaging conditions like consistent lighting, fixed camera distance and angle, use tongue depressors or retractors for a clear view, ensure tongue is clean and well-hydrated. Capture pictures from multiple angles without any reference images.

#### Metadata collection

For every image, record accompanying metadata like Size, colour, texture, location, surface pattern, Age, sex, ethnicity, Device used, resolution, lighting condition, etc.

#### Data cleaning

**Fig. 3:**
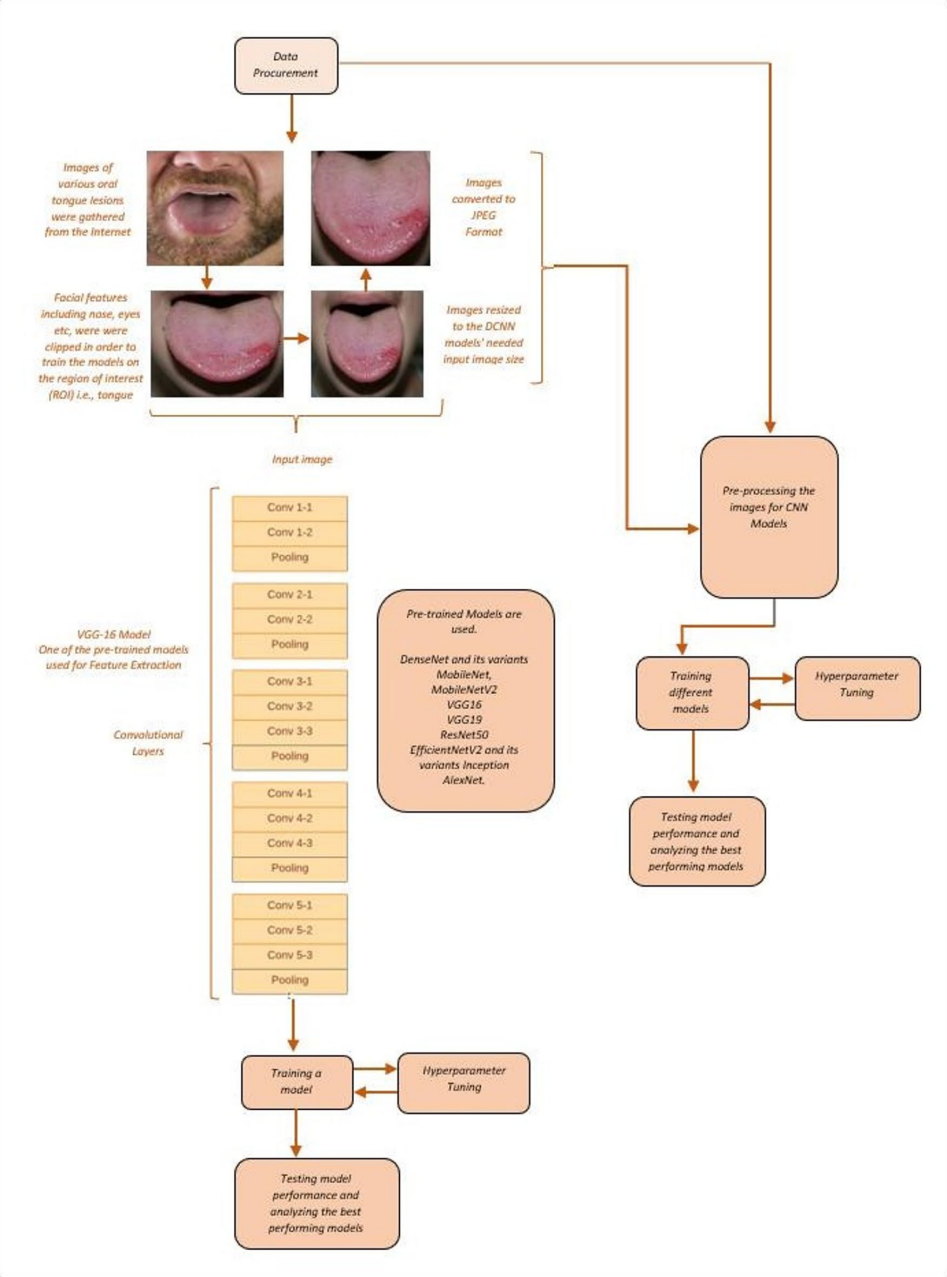
Flow chart of the proposed system.

After raw data collection, de-identification, such as removing any identifiable features (faces, patient IDs, timestamps) and renaming images using unique anonymized IDs. Consider image quality assessment, such as eliminating blurred, poorly lit, or obstructed images, and normalizing brightness/contrast, if necessary, crop to focus on the lesion area using manual annotation. Remove irrelevant background or anatomical structures using annotation tools such as label box and VGG image annotator (VIA), and finally split the dataset into training, validation, and testing sets. However, a cleaned, standardized, and annotated image dataset of oral tongue lesions is obtained for further processing, as shown in Fig. 3.

The Fig. 3 illustrates that photographs of oral tongue lesions were collected online using an image search engine. The training courses and lesion pictures were annotated manually. Subsequently, each image was converted to the JPEG format required by the deep neural network. Before the model training phase, they reduced the required input picture size for the DCNN models. Facial characteristics such as the nose, eyes, and other unnecessary elements of the photos were truncated to train the models on the region of interest (ROI), namely the tongue, to enhance model accuracy. This approach also anonymised the photos to protect patient privacy. **Input Images**: The input photos were sourced from a custom-developed OC Dataset. The photos are already categorized into training and validation directories. The training folder contains 280 photos, whereas the validation folder has 94 images.

This research examined several pre-trained CNNs, including DenseNet and its variant, MobileNet, MobileNetV2, VGG16, VGG19, ResNet50, EfficientNetV2, along with its variant, Inception, and AlexNet. A dense network (DenseNet) interlinks each layer in a feed-forward manner. It resolves the vanishing-gradient problem, improves component transmission, increases feature reuse, and significantly decreases the parameter count. DenseNet works by cutting down on connections between layers close to the input and those close to the output. It teaches convolutional networks at deeper levels, improving their accuracy and efficiency. The image from the original study, shown here, offers an elegant representation of scaling. DenseNet is available in many variations, including DenseNet121, DenseNet169, and DenseNet20.

## Results and discussion

All experiments were conducted on Google Colab with GPU acceleration, which provides a consistent cloud-based environment for deep learning. The efficacy of the DCNN models was evaluated on the task of binary classification of tongue lesions (malignant vs. non-cancerous). Model development and training were carried out using TensorFlow and Keras libraries, with the Keras functional API applied to initialize pre-trained models and extract input feature vectors. NumPy and OpenCV were employed for image preprocessing, while data augmentation techniques (e.g., flips, rotations, and brightness adjustments) were used to improve generalization.

All models, namely DenseNet121, DenseNet169, DenseNet201, MobileNet, MobileNetV2, VGG16, VGG19, ResNet50, EfficientNetV2B0, EfficientNetV2B1, EfficientNetV2B2, EfficientNetV2B3, Inception, and AlexNet, were trained on identical data while maintaining consistent hyperparameters, except the optimizers, for a duration of 20 epochs. The models were constructed independently with three optimizers: RANGER, ADAM, and SGD, and the accuracy and loss were recorded are given in Table 1. The epochs were minimized to identify the optimal model that could be trained within a reduced timeframe. The lesion data about the mouth cavity is currently inaccessible. No pre-existing tongue dataset was available for this study. The dataset analyzed here was compiled by the authors from non-licensed, clinically annotated photos collected from publicly available internet sources and public repositories.

**Table 1:** Comparison of different optimizers accuracy and loss.

Type of optimizer	RANGER		ADAM		SGD	
Filed	Training	Validation	Training	Validation	Training	Validation
Inception with 20 Epochs						
Accuracy	0.8333	0.8167	0.8500	0.8167	0.7500	0.7500
Loss	0.4605	0.3752	0.3981	0.5125	0.5239	0.6344
Densenet 121 with 20 Epochs						
Accuracy	0.8516	0.7344	0.9766	0.8906	0.9844	0.8750
Loss	0.3270	0.5145	0.1079	0.3165	0.2421	0.4083
Densenet 169 with 20 Epochs						
Accuracy	0.8583	0.8594	0.9766	0.8906	0.9844	0.8750
Loss	0.3648	0.4264	0.1079	0.3165	0.2421	0.4083
Densenet 169 with 20 Epochs						
Accuracy	0.8917	0.7656	0.9766	0.8906	0.9844	0.8750
Loss	0.3743	0.5081	0.1076	0.3165	0.2421	0.4083
Mobile Net with 20 Epochs						
Accuracy	0.9917	0.65620	0.9766	0.8906	0.9844	0.8750
Loss	0.0458	1.4840	0.1079	0.3165	0.2421	0.4083
Mobile NetV2 with 20 Epochs						
Accuracy	0.9833	0.8281	0.9766	0.8906	0.9844	0.8750
Loss	0.0780	0.3934	0.1079	0.3165	0.2421	0.4083
VGG16 with 20 Epochs						
Accuracy	0.9766	0.8906	0.9766	0.8906	0.9844	0.8750
Loss	0.0477	0.2654	0.1079	0.3165	0.2421	0.4083
VGG19 with 20 Epochs						
Accuracy	0.9250	0.8000	0.8750	0.8000	0.6000	0.6750
Loss	0.3031	0.4896	0.3918	0.4970	0.6472	0.5959
ResNet50 with 20 Epochs						
Accuracy	0.8906	0.2344	0.9766	0.8906	0.9844	0.8750
Loss	0.2799	0.7971	0.1079	0.3165	0.2421	0.4083
EfficientNetV2B0 with 20 Epochs						
Accuracy	0.6641	0.6250	0.9766	0.8906	0.9844	0.8750
Loss	0.5838	0.6689	0.1079	0.3165	0.2421	0.4083
EfficientNetV2B1 with 20 Epochs						
Accuracy	0.6641	0.3125	0.9766	0.8906	0.9844	0.8750
Loss	0.5740	0.8009	0.1079	0.3165	0.2421	0.4083
EfficientNetV2B2 with 20 Epochs						
Accuracy	0.6562	0.6179	0.9766	0.8906	0.9844	0.8750
Loss	0.6258	0.6307	0.1079	0.3165	0.2421	0.4083
EfficientNetV2B3 with 20 Epochs						
Accuracy	0.6406	0.7344	0.9766	0.8906	0.9844	0.8750
Loss	0.6240	0.6123	0.1079	0.3165	0.2421	0.4083
Alex Net with 20 Epochs						
Accuracy	0.8667	0.7167	0.8833	0.6000	0.7000	0.7167
Loss	0.3892	0.7727	0.3724	0.8907	0.6975	0.6748

The compiled dataset is available from the corresponding author upon reasonable request. As a result, a distinctive dataset was generated using clinically annotated photos. The photos were categorized into malignant and non-malignant categories. The dataset included 374 photos, including 126 malignant and 248 non-cancerous specimens. Of these, 280 images (75%) were allocated for model training, while 94 images (25%) were designated for validation. The results were used to compare with accuracy measures, which are often used to provide equal significance to all classes. The dataset used in this study was balanced, with both classes exhibiting equivalent critical accuracy measures and established reliability.

$${\text{Accuracy}} = \left( {{\text{True Positive}} + {\text{True Negative}}} \right)/\left( {{\text{True Positive}} + {\text{False Positive}} + {\text{True Negative}} + {\text{False Negative}}} \right).$$

**Fig. 4:**
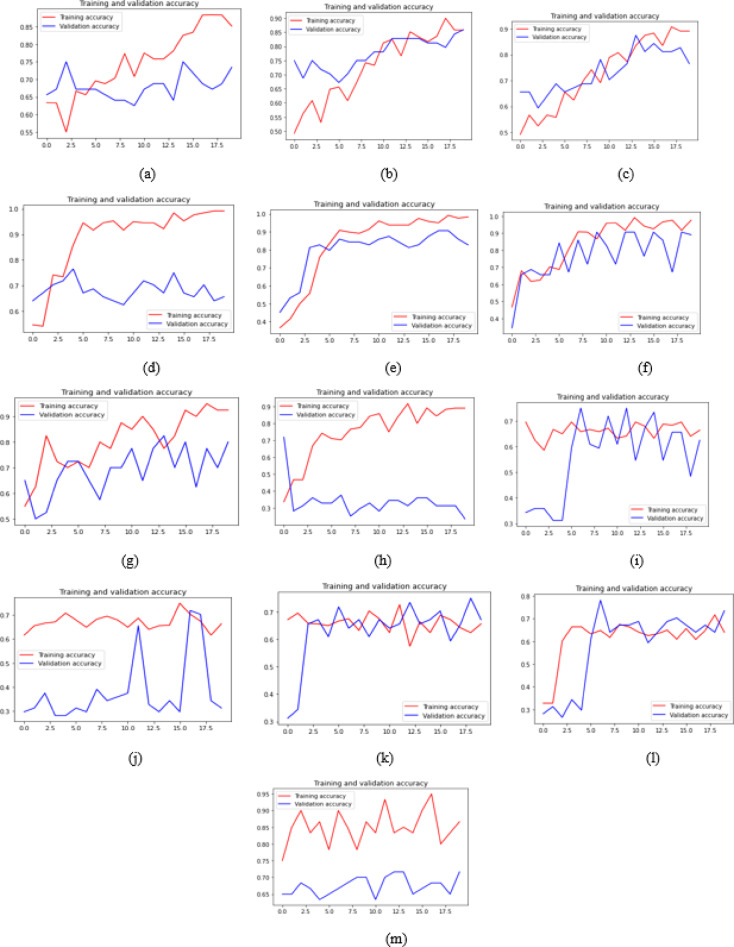
Accuracy Plot for (**a**) Inception Model (**b**) DenseNet169 (**c**) DenseNet169 (**d**) MobileNet (**e**) MobileNetV2 (**f**) VGG16 (**g**) VGG19 (**h**) ResNet50 (**i**) EfficientNetv2B0 (**j**) EfficientNetv2B1 (**k**) EfficientNetv2B2 (**l**) EfficientNetv2B3 (**m**) AlexNet1.

**Fig. 5:**
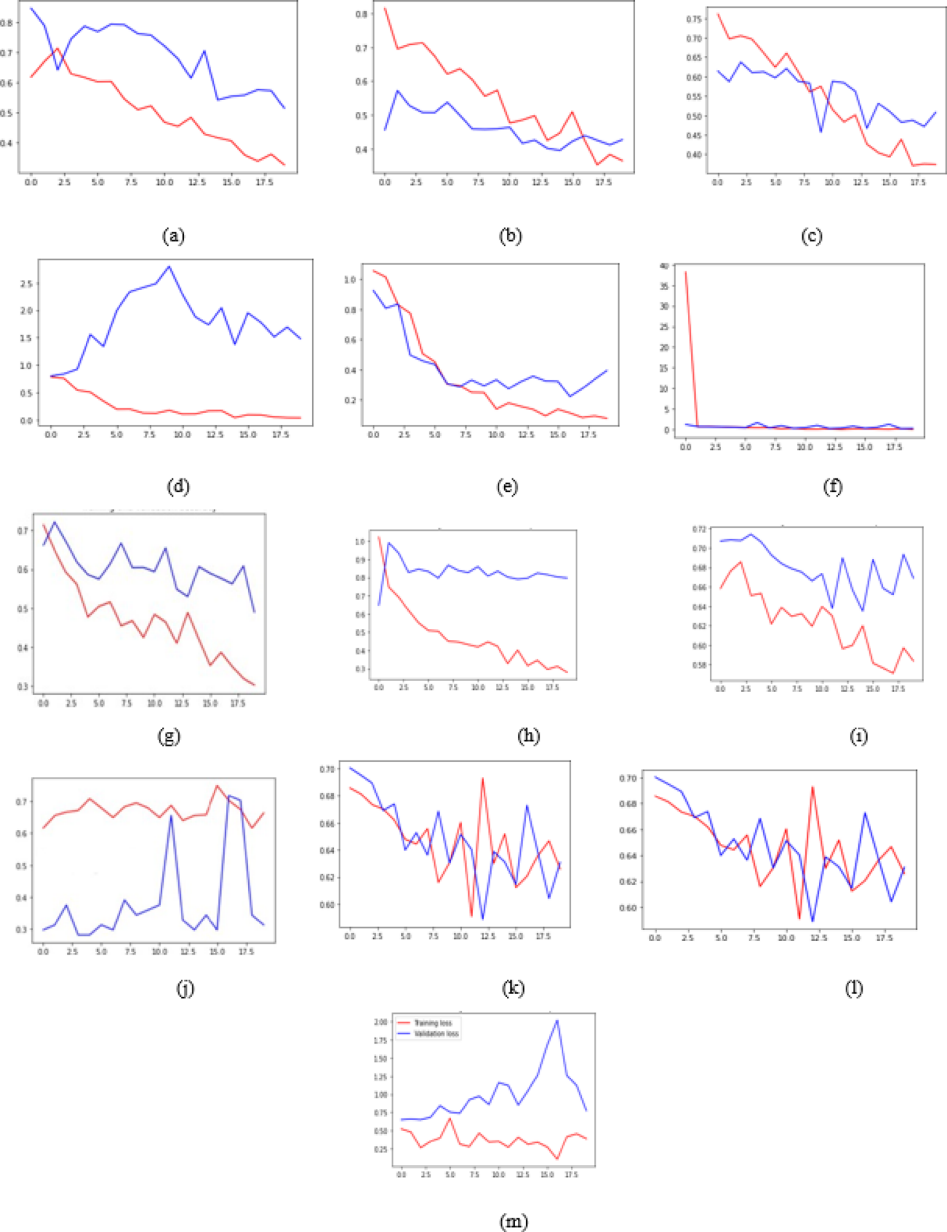
Loss Plot for (**a**) Inception Model (**b**) DenseNet169 (**c**) DenseNet169 (**d**) MobileNet (**e**) MobileNetV2 (**f**) VGG16 (**g**) VGG19 (**h**) ResNet50 (**i**) EfficientNetv2B0 (**j**) EfficientNetv2B1 (**k**) EfficientNetv2B2 (**l**) EfficientNetv2B3 (**m**) AlexNet1.

Two distinct line graphs were generated. The first graph illustrates the accuracy, comparing training and validation accuracy is given in Fig. 4. The second graph illustrates a comparison between training loss and validation loss is given in Fig. 5. Observations were recorded based on accuracy and loss. To enhance model interpretability, many photos were inputted into the model, which classified them as malignant or non-cancerous according to the sigmoid output percentage for each class.

**Table 2:** Comparison of different approaches training and validation.

Type of optimizer	RANGER		ADAM	
Filed	Training	Validation	Training	Validation
Densenet 121	0.8516	0.7344	0.3270	0.5145
Densenet 169	0.8583	0.8594	0.3648	0.4264
Densenet 201	0.8917	0.7656	0.3743	0.5081
Mobile Net	0.9917	0.6562	0.0458	1.4840
Mobile NetV2	0.9833	0.8281	0.0780	0.3934
VGG16	0.9766	0.8906	0.0477	0.2654
VGG19	0.9250	0.8000	0.3031	0.4896
ResNet50	0.8906	0.2344	0.2799	0.7971
EfficientNetV2B0	0.6641	0.6250	0.5838	0.6689
EfficientNetV2B1	0.6641	0.3125	0.5740	0.8009
EfficientNetV2B2	0.6562	0.6719	0.6258	0.6307
EfficientNetV2B3	0.6406	0.7344	0.6240	0.6123
Inception	0.8333	0.8167	0.4605	0.3752
Alex Net	0.8667	0.7167	0.3892	0.7727

As shown in previous studies, the DCNN model performs superiorly to ANN and ML models. Although ANN performs nearly as well as DCNN, DCNN models remain superior due to their enhanced capability to discern features autonomously by evaluating the comprehensive outcomes of each categorization model. The results indicate that DCCN methodologies are dependable for identifying oral cancer via tongue lesions using CNN algorithm.

From Table 2, Considering the Ranger optimizer, MobileNet has achieved a peak training accuracy of 99.17%. The maximum validation accuracy recorded is 89.06% for VGG16. VGG16 exhibits the lowest training loss of 0.0477 and the lowest validation loss of 0.2654. The training accuracy of VGG16 is commendable at 97.66%. The validation accuracy of MobileNet is 65.62%, while the validation loss is 1.480. So, VGG16 did better on the used dataset, with a training accuracy of 97.66%, after looking at all metrics, such as training accuracy, validation accuracy, training loss, and validation loss. The following model, MobileNetV2, outperformed the other models in terms of training accuracy, validation accuracy, training loss, and validation loss, with a training accuracy of 98.33%, a validation accuracy of 82.81%, a training loss of 0.0780, and a validation loss of 0.3934. The models identified as underperforming are EfficientNetV2B0, EfficientNetV2B1, EfficientNetV2B2, and EfficientNetV2B3.

## Conclusion and future work

This study discusses many deep-learning classification algorithms for ovarian cancer diagnosis. The results of several classifiers for automated early diagnosis of ovarian cancer have been presented. The research is distinctive in that it only examines lesions that arise on the tongue. The promising model results demonstrate the efficacy of deep learning and suggest its potential to manage these challenging jobs. Due to the absence of a dataset for tongue lesions in the oral cavity, the study utilizes a uniquely developed dataset, which is innovative. This study used many CNN architectures: DENSENET, MOBILENET, VGGNET, RESNET, EFFICIENTNET, INCEPTION, and ALEXNET. The aggregate accuracy of CNN classifiers is 97.21%. The results indicate that the CNN method is proficient in identifying cavity cancer. The findings suggest that VGG16 attains the highest rate among the different techniques, with a training accuracy of 97.66%. The findings presented above include limitations. Aside from tongue lesions and instances necessitating further biopsy analysis, the model cannot substitute for human pathology assessments. Given its proximity to the tongue, more research is necessary to develop DCNNs for categorizing activities occurring in other regions of the oral cavity, such as the lips or the inner side of the cheeks. The model delineated herein is designed for tongue lesions; however, due to their external similarities, it may also be utilised for lesions in other regions of the oral cavity. The model trained on imaging data of tongue lesions to find dysplastic abnormalities may be able to tell which lesions are likely helpful in other parts of the mouth. This initiative represents progress. Future endeavours will enable acquiring more photos to augment the dataset and improve the models’ accuracy using diverse tactics. The main goal is to execute semantic segmentation to identify lesion regions in an input picture to enhance model accuracy results. Moreover, the research may be expanded to address other stages of cancer.

## Data Availability

The datasets generated and analyses during the current study are not publicly available because clinically annotated photos collected from publicly available internet sources and public repositories , but are available from the corresponding author on reasonable request.
